# Personal and Societal Health Quality Lost to Tuberculosis

**DOI:** 10.1371/journal.pone.0005080

**Published:** 2009-04-08

**Authors:** Thaddeus L. Miller, Scott J. N. McNabb, Peter Hilsenrath, Jotam Pasipanodya, Stephen E. Weis

**Affiliations:** 1 School of Public Health, University of North Texas Health Science Center at Fort Worth, Ft. Worth, Texas, United States of America; 2 Department of Medicine, University of North Texas Health Science Center at Fort Worth, Ft. Worth, Texas, United States of America; 3 Division of Integrated Surveillance Systems and Services, National Center for Public Health Informatics, Centers for Disease Control and Prevention, Atlanta, Georgia, United States of America; 4 Tarrant County Public Health Department, Ft. Worth, Texas, United States of America; Stanford University, United States of America

## Abstract

**Background:**

In developed countries, tuberculosis is considered a disease with little loss of Quality-Adjusted Life Years (QALYs). Tuberculosis treatment is predominantly ambulatory and death from tuberculosis is rare. Research has shown that there are chronic pulmonary sequelae in a majority of patients who have completed treatment for pulmonary tuberculosis (PTB). This and other health effects of tuberculosis have not been considered in QALY calculations. Consequently both the burden of tuberculosis on the individual and the value of tuberculosis prevention to society are underestimated. We estimated QALYs lost to pulmonary TB patients from all known sources, and estimated health loss to prevalent TB disease.

**Methodology/Principal Findings:**

We calculated values for health during illness and treatment, pulmonary impairment after tuberculosis (PIAT), death rates, years-of-life-lost to death, and normal population health. We then compared the lifetime expected QALYs for a cohort of tuberculosis patients with that expected for comparison populations with latent tuberculosis infection and without tuberculosis infection. Persons with culture-confirmed tuberculosis accrued fewer lifetime QALYs than those without tuberculosis. Acute tuberculosis morbidity cost 0.046 QALYs (4% of total) per individual. Chronic morbidity accounted for an average of 0.96 QALYs (78% of total). Mortality accounted for 0.22 QALYs lost (18% of total). The net benefit to society of averting one case of PTB was about 1.4 QALYs.

**Conclusions/Significance:**

Tuberculosis, a preventable disease, results in QALYs lost owing to illness, impairment, and death. The majority of QALYs lost from tuberculosis resulted from impairment after microbiologic cure. Successful TB prevention efforts yield more health quality than previously thought and should be given high priority by health policy makers. (Refer to Abstracto S1 for Spanish language abstract)

## Introduction

Paradoxically, success in TB prevention and control can result in impediments that threaten elimination efforts [1]. Historically, as incidence rates fell, funding for TB prevention and control was reduced and costs—per TB case averted—rose. Increasing TB costs compromised the ability of public health authorities to justify TB prevention activities for other public health programs. As a result, TB managers experienced increased pressure to justify support of the TB public health practice infrastructure. And we know now, that past failure to maintain support for TB public health practice infrastructure leads to TB resurgence [2]–[4].

The appropriate use of limited government resources requires accountability for health effects (i.e., reduction of both morbidity and mortality and of health disparities). The Quality-Adjusted Life Year (QALY) is a common measure for comparing health effects between interventions. This measure represents the relative value of a year-of-life after weighting for quality effects [5]. Scaled between 0 (worst health) and 1 (best health), QALYs allow broad economic comparisons between widely disparate outcomes (e.g., highway building, OSHA regulations, public health programs, medical procedures) [5], [6]. Ideally, the use of such measures enables policy makers to improve economic efficiency by directing resources to activities that yield the greatest health effect per unit of cost. Further, complete and accurate estimations of health effects require that health outcomes be fully measured; this includes QALYs.

In the past, TB health effects have not been fully measured. In low-incidence areas—while empirically recognized as an important public health problem—TB is not considered to result in substantial loss of quality of life. TB is curable, and with a relatively short, well-tolerated, and inexpensive treatment it has relatively low mortality rates in the developed world [7]. As a result, most TB patients experience limited illness under an ambulatory course of treatment. In these circumstances, QALYs lost to acute illness and ambulatory treatments are minimal. So, it has been argued that TB cure may be substituted for prevention as an efficient policy option when the cost to prevent a TB case exceeds the cost of its cure [8], [9]. However, this judgment should be made only when benefits are estimated, including the relevant cost for the value of social benefits and using appropriate discounting. In fact, all costs associated with TB have not been measured, and current policy judgments are not completely informed.

Although many TB health effects are well categorized, key gaps do exist. For example, we recognized that some TB patients with microbiologically cured disease do become disabled, but the effect of this chronic morbidity has not been measured [10], [11]. Further, measures of acute TB illness and treatment, continuing impairment after cure, and death resulting from TB are important components of the full cost to society [12]–[14].

We estimated societal QALYs lost to TB. Our analyses included studies of the health effects of acute illness, treatment, pulmonary impairment after tuberculosis (PIAT), and death, enabling a more nearly complete estimate of the effects of TB to society.

## Methods

We estimated the lifetime QALYs for individual TB patients with culture-confirmed pulmonary disease and the aggregated QALYs lost to prevalent TB disease in Tarrant County, Texas, during 2002 [10], [11]. We based estimates in part on published data from cohorts with tuberculosis and latent tuberculosis infection (LTBI) [10], [11]. Cohort demographics and measures of health after treatment were taken from the same studies (table 1) [10], [11]. Values for health during illness and treatment, death rates, years of life lost to death, and normal population health were taken from previously published sources (table 2). A decision model was designed using TreeAge Pro 2005 (1075 Main Street, Williamstown, MA 01267) for each of three states: normal health; latent tuberculosis infection; and culture confirmed tuberculosis. This model, along with other calculations to be described later, was used to derive and compare estimates of the lifetime expected QALYs for each of the three states.

**Table 1:** Characteristics of patients treated for pulmonary tuberculosis (cases) and LTBI (comparison), 2002, Tarrant County, Texas [10].

Characteristics	Cases (N = 107) (%)	Comparison (N = 210) (%)	p-value
Male	74(69)	111(53)	0.005**
Caucasian	25(23)	47(22)	0.889
Hispanic	29(27)	55(26)	0.943
African-American	23(22)	55(26)	0.407
AAIO	30(28)	53(25)	0.523
Ever smoked	61(57)	107(51)	0.217
Ever used crack cocaine	21(20)	36(17)	0.395
Occupational pulmonary risk	6(6)	14(7)	0.834
US-born	55(51)	111(53)	0.831
HIV positive	15(14)	15(7)	0.186
Not Done		24(11)	
Pulmonary impairment	63(59)	41(20)	<0.001**

	Mean (SD)	Mean (SD)	
Age (years)	47(15)	41(15)	0.003##
BMI	23.75(4.98)	27.66(5.78)	<0.001##
Height (in)	67(4)	66(4)	0.163
(Smoking) pack-years	12.01(18.24)	7.24(14.36)	0.011##
FVC (% predicted)	82.77(24.89)	95.51(19.49)	<0.001##
FEV1 (% predicted)	77.77(27.37)	95.43(19.48)	<0.001##
FEV1/FVC (% predicted)	76.07(13.34)	83.97(7.85)	<0.001##
FEF 25–75 (% predicted)	72.68(41.45)	98.61(31.79)	<0.001##

Table used with permission.

Abbreviations: AAIO-Asians, Africans, Indian, Other; FVC-Forced Vital Capacity; FEV1-Forced Expiratory Volume in One Second; FEF 25-75-Mid Forced Expiratory Flow.

** p<0.05 based on Pearson Chi-square.

## t-test.

**Table 2:** Values and weights used in the estimation of health quality lost, 2002, Tarrant County, Texas.

Values	Weight	Source
Health state valuation weight for illness, convalescence, and treatment: nonfatal TB	0.9	Mauchand, 1999
Health weight s/p pulmonary tuberculosis, no identified pulmonary impairment*	0.921	Pasipanodya, 2007
Health weight s/p pulmonary tuberculosis, mild pulmonary impairment*	0.837	Pasipanodya, 2007
Health weight s/p pulmonary tuberculosis, moderate pulmonary impairment*	0.81	Pasipanodya, 2007
Health weight s/p PTB patient, severe pulmonary impairment*	0.694	Pasipanodya, 2007
Average health weight s/p PTB, all survivors*	0.86	Pasipanodya, 2007
Baseline health state valuation adjusted matched to cohort demographics	0.89	WHO, 2003
Years of life lost to tuberculosis death (in hospital)	16.6	Hansel, 2004; CDC, 2002
TB death rate	0.047	CDC, 2005

### Literature Reviewed

We searched OVID from 1950 to 2007 by combining keyword “tuberculosis” with each of the modifiers “quality-adjusted life years,” “health status,” “disability,” “cost of illness,” and “disability-adjusted life years.” The search was limited to the English language, full-text results, and we reviewed all previously reported studies of the health status of persons with PTB. We found no societal estimates of health quality lost to TB in this search.

### Weighting Effects of TB

Weights giving the health effects of time spent with acute illness and the effect of chemotherapy for the treatment of TB, including adverse drug interactions, have been estimated, as have health state valuations for geographic regions and average losses due to death during acute illness (table 2) [15]–[19]. Pulmonary impairment after TB (PIAT) is a chronic sequela, and its health effect has been identified but not weighted [10], [11]. The health effect for the sequelae of TB was estimated by combining studies of PIAT with the American Medical Association's (AMA) pulmonary grading criteria to determine the degree of impairment. Impairment was graded as none, mild, moderate, or severe [10], [20]–[22]. Grades of impairment were matched with quantitative measures of health quality, measured by the St. George's Respiratory Questionnaire (SGRQ) [11]. The average health weight for each grade of impairment was calculated as the percentage difference in SGRQ score from that found in persons with similar risk factors, and a weighted average health weight for all pulmonary tuberculosis patients was calculated.

### Sources of Health Lost

We assumed that differences in the cumulative expected QALYs lost by cohorts with and without TB were due to acute illness and treatment, chronic health loss (impairment) after treatment, and death. All individual and cohort demographics were based on 107 PTB patients participating in two Tarrant County studies (table 1). Baseline health state valuation was then adjusted to age, sex, and region [17]–[19].

### QALYs Lost Owing to Acute Illness and Treatment

The duration of acute illness and treatment were conservatively estimated as six months, and the outcome is assumed to be cure or death. Time spent ill and in treatment was multiplied by the baseline health valuation for each individual and the weight for illness and ambulatory treatment. The total number of QALYs that accrued to the cohort as a unit during that time is expressed as a sum for the cohort and as an average for individual members of the cohort. The QALYs lost to TB during this time is assumed to be the difference between these values and those obtained from the same groups without weighting for illness and treatment.

### QALYs Lost Owing to Impairment

We assumed that pulmonary function in PTB patients after 20 weeks of treatment would remain stable for the remainder of the patient's life [23]–[25]. We also assumed that health lost following treatment would continue throughout the patient's lifetime. To estimate the years lived with disability, we used life tables adjusted for sex, age, and geographic region, and we estimated the years of life lived from the time of microbiologic cure to predicted death [19]. Time spent from the end of treatment until death was multiplied by the baseline health valuation and the weight for each patient's impairment. Total QALYs lost to the cohort as a whole during that time was expressed as a sum for the cohort and as an average for individual members of the cohort. QALYs lost to PTB during this time were assumed to be the difference in total lifetime QALYs expected if illness had not occurred.

### QALYs Lost to Death

We assumed that QALYs lost to death began to accrue from before or during acute illness. However data related to deaths before and after enrollment were not collected for this cohort [10]. We therefore estimated cohort deaths, based upon national data [18]. QALYs lost to death from TB were estimated based on prior reports giving the average age and the sex of persons who had died from acute TB [16], [19]. We adjusted life tables for sex, age, and geographic region and used these to estimate years of life remaining until expected natural death [19]. QALYs lost to death from TB were then estimated as the difference in total lifetime QALYs expected had death not occurred. We did not include an estimation of QALYs lost owing to excessive deaths following completion of treatment.

### Health Quality Calculations

We estimated lifetime health quality for persons without TB by calculating the baseline health quality (BHQ) for an age range multiplied by the number of years spent in that state; then we summed over the expected natural life. For patients with microbiologically cured PTB, the baseline was adjusted to reflect health quality lost to illness, treatment, and impairment. One-time quality losses to acute illness and TB treatment (QLTBT) were then subtracted. Weighting for the health quality effect of pulmonary impairment after TB (QPIAT) began at its identification and continued for subsequent expected years of life. This expression took the form (BHQ*time in state*QPIAT) - QLTBT = lifetime QALYs for an individual status post PTB. To estimate the QALYs lost to all patients with PTB, the same formula was used but included one-time losses for death during acute illness.

### Statistical Analyses

We used SPSS, version 11.0 for Windows (SPSS Inc. 233 S. Wacker Drive, 11th floor, Chicago, IL 60606-6307) to perform statistical calculations. Significant differences between groups were determined by using the Student *t*-test and the *x* ^2^ test. Calculations relating to health quality, discounting, and weighting were performed using Microsoft Excel 2002 (Microsoft Corporation, Redmond, WA). QALYs were reported as net of social discount rates that were varied from 3%–8% per annum for predicted the lifespan [26]–[28]. Discounting captured the inter-temporal opportunity cost-of-capital and the rate-of-time preference for the present over the future. Unless otherwise stated, findings were reported net of a 3% social discount rate.

## Results

We estimated the health quality of the entire cohort of 107 PTB patients. Pulmonary function tests showed that 63 (58.9%) of PTB patients had pulmonary impairment as defined by the AMA (table 2). Thirty-seven (34.6%) patients suffered mild; 15 (14.0%) moderate; and 11 (10.3%) suffered severe impairment (table 3). Forty-one of 210 (19.5%) patients with LTBI had some pulmonary impairment. The average severity of impairment was less in those patients with LTBI, with 35 (16.7%) having mild; 5 (2.4%) moderate; and 1 (0.5%) patient suffering severe pulmonary impairment (table 3).

**Table 3:** Proportion of pulmonary impairments among pulmonary tuberculosis (cases) and control cohorts‡, 2002, Tarrant County, Texas.

Impairment†	Cases				all cases		Controls				all controls	
	male		female				male		female			
	n	%	n	%	n	%	n	%	n	%	n	%
None	25	0.34	18	0.53	43	0.4	89	0.81	77	0.79	166	0.8
Mild	27	0.37	10	0.29	37	0.35	19	0.17	16	0.16	35	0.17
Moderate	13	0.18	2	0.06	15	0.14	2	0.02	3	0.03	5	0.02
Severe	8	0.11	4	0.12	12	0.11	0	0	1	0.01	1	0
total	73	1	34	1	107	1	110	1	97	1	207	1

‡ Differences in proportion significant at 0.05 level.

† Impairment level defined as <10% difference from normative = no impairment; 10–25% = mild; 26–50% = moderate; 51–100% = severe.

### QALYs Lost to Acute Illness and Treatment

For the entire cohort of 107 PTB patients, we estimated that acute illness and treatment resulted in a total of 5.35 QALYs lost. Each individual PTB patient averaged a loss of 0.046 QALYs prior to clinical cure (table 4).

**Table 4:** Quality-adjusted life years lost associated with pulmonary tuberculosis, 2002, Tarrant County, Texas.

Source	Gross	Net 3% Discount
QALY losses to cohort		
TB death*	69.6	24.3
Acute illness/treatment*	5.35	n/a
Disability	294.25	102.73
Total QALY loss to cohort*	369.2	128.90***
QALY losses to individuals		
Total per TB patient*	3.3	1.39***
Average loss prior to clinical cure per pt	0.046	n/a
Average loss to death*	0.621	0.38
Total per PTB survivor**	2.8	0.98
Average loss to disability d/t PTB	2.75	0.96
Estimated QALYs lost to TB death, per death	13.92	8.52

* N = 112, assumes 5 deaths prior to completion of treatment.

** N = 107.

*** Discounting considers: no discount for acute illness/treatment; 16.6 years for early death, and 35.6 years to expected natural death for survivors.

### QALYs Lost to Impairment

We estimated that, following therapy, the entire PTB cohort had lost a net total of 102.73 QALYs. Averaged across the PTB cohort, individuals would lose a net total 0.96 QALYs (table 4).

### QALYs Lost to Death

Assuming an average TB death rate of 4.5%, we estimated that 5 TB patients would die prior to entering the cohort. These deaths would then account for a total loss of 69.6 undiscounted or 24.30 discounted QALYs (table 4). Every individual TB death resulted in a net loss of 8.52 QALYs (table 4).

### Lifetime expected QALYs

We estimated the lifetime expected QALYs for each group (table 5). Normal populations (weighted to the age and demographics of the study cohort with a history of PTB), had a lifetime expectation of 69.11 QALYs. We predicted that cohort members with LTBI could expect 67.43 QALYs; and that those with treated PTB could expect 64.68 QALYs over their life spans (table 5). We predicted that persons with LTBI could expect 1.69 fewer undiscounted QALYs than persons in the normal population. We predicted that persons with a history of PTB could expect 4.43 fewer QALYs than persons in a normal population and 2.75 fewer QALYs than those in the LTBI group (table 5). From the time of cure to their expected death, patients with a history of PTB can expect an average of 0.98 QALYs less than the patients in the LTBI group.

**Table 5:** Expected lifetime QALYs lost, by condition, Tarrant County, Texas, 2002.

Condition	QALYs	% normal	QALY difference from normal	QALY difference from LTBI
Population normal *	69.11	100%	0	n/a
Normal+LTBI	67.43	97.56%	1.69	0
Normal+microbiologically-cured PTB**	64.68	93.59%	4.43	2.75

* Expected, weighted to sex/age of cohort.

** Estimates based on survivors only.

For patients diagnosed with PTB during the study period, we estimated an expected difference of 128.9 fewer lifetime QALYs from the same cohort weighted to reflect health loss of persons with LTBI. Illness and treatment accounted for 1.5%; death for 18.9%; and PIAT for 79.8% of the total QALY difference (table 4).

On average, an individual difference of 1.2 net QALYs was lost between those who have had PTB and those without (but with similar risks). The greatest share of this difference—0.96 QALYs—came from disability after successful treatment. A total average difference of 1.4 fewer discounted QALYs was expected to accrue to patients that acquired PTB than those with LTBI. This measure reflects the savings possible through prevention of one case of PTB.

Sensitivity analyses of discount rates gave QALY loss from TB morbidity and mortality a range from 3.3 at 0% discount to 0.35 at an 8% discount per annum.

## Discussion

In this study, we analyzed—for the first time—the health quality loss related to PTB. We estimated the total QALYs lost to illness, treatment, pulmonary sequelae, and death related to PTB. We compared the expected lifetime QALYs for patients with treated PTB to that expected to accrue to a normal population, weighted to the age, sex, and location of the cohorts studied; and to QALYs expected for a population with LTBI. Although patients with LTBI share many risk factors with those having PTB, they do not experience acute disease and its associated effects. We estimated an average of 69.11, 67.43, and 64.68 lifetime QALYs would accrue to normal, LTBI, and PTB populations, respectively. These data show that, on average, persons microbiologically cured of TB have suffered an important, preventable health loss. Our data also show that most health losses from TB occur after microbiologic cure of disease; such losses have previously been unmeasured. The net benefit of averting one case of PTB is at least 1.4 QALYs. Further, these data illustrate that successful treatment of LTBI yields substantially more population health than previously recognized.

While PIAT is a chronic common sequela of PTB, we found no prior estimates of health lost to PTB that includes it. We estimated that PIAT accounts for a difference of 0.96 fewer QALYs in persons treated for PTB when compared to persons with similar risks. QALYs lost related to PIAT comprise 80% of the total QALYs difference between groups. These data demonstrate that previous studies of health outcomes of PTB have considerably underestimated the full societal effects of PTB.

These data also suggest that treatment of LTBI has important benefits not considered in the design of the current treatment guidelines. The knowledge that chronic PTB sequelae occur frequently despite successful treatment of PTB may influence clinicians to recommend treatment of LTBI with greater frequency. In addition, patient acceptance of LTBI treatment may be enhanced by the potential benefit of preventing chronic lung disease. The resulting health benefits may be substantial; we found that a history of PTB yields a health weight of 0.857, an effect far greater than that reported for many chronic conditions (table 6) [13], [29], [30]. For population health, QALYs lost to survivors with pulmonary impairment are 4.4 times greater than QALYs lost to PTB mortality.

**Table 6:** Relative effect of chronic illness on individuals†, Tarrant County, Texas, 2002.

Condition	Weight
Alcohol or drug related problems	0.768
Anxiety/depression	0.791
Other health problems	0.807
Diabetes	0.835
Arms, legs hands, feet, back or neck	0.841
Epilepsy	0.841
Stomach/liver/kidneys	0.851
Chest, breathing problems, asthma, bronchitis	0.857
Microbiologically cured Pulmonary Tuberculosis‡	0.86
Heart, blood problems, or circulation	0.875
Migraine or frequent headaches	0.934
Difficulty in seeing	0.938
Difficulty in hearing	0.962
Skin conditions/allergies	0.966

† Adapted with permission from: Happiness Quantified, Van Praag, Ferrer-i-Carbonell 2004 (based on self-reported chronic conditions in Britain and self-assessment of effects).

‡ As found, survivors of TB completing standard therapy.

These data demonstrate that the treatment of LTBI has been undervalued in the past and that future LTBI treatment guidelines should include consideration of the benefit of disability prevention [31]. This may result in recommending treatment of LTBI in persons currently considered at lower risk for progressing to TB.

**Figure 1:**
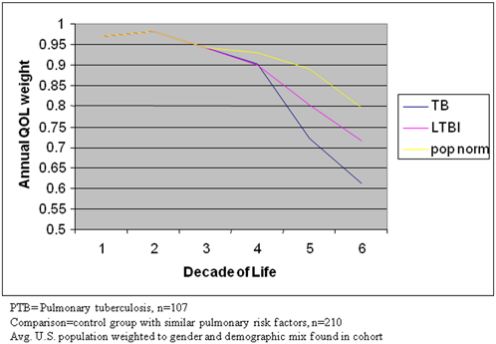
By decade of life and cohort, the annual quality of life, 2002, Tarrant County, Texas. PTB = Pulmonary tuberculosis, n = 107. Comparison = control group with similar pulmonary risk factors, n = 210. Avg. U.S. population weighted to sex and demographic mix found in cohort.

These data also have implications for global health policy. Most PTB cases occur outside the United States, many in economies with insufficient medical infrastructure [32]. In the population studied, the burden of disability begins at midlife and continues until death, corresponding not only with peak earning years but also during a period of child bearing and rearing and elder care (figure 1) [33]. By addressing an important, preventable cause of disability in these populations through strengthening TB prevention programs, it may be possible to effect significant economic and social gains as well as gains to individual health.

The discounting of future health benefits is controversial [26]–[28]. Discounting the monetary value of QALYs is well accepted and expected for studies where cost and health outcomes are spread across long periods. But the discounting of health relies on the assumption that there is preference for health in the present over the future, a presumption that is supported by observations of behaviors from smoking to dietary habits [26]–[28]. When discount rates are higher or when benefits occur far in the future, the present value of future savings is lower. The present value of Quality-Adjusted Life Years in this analysis remains substantial even at a discount rate of 8% over almost 36 years, the average expected years of life remaining for the cohort and the longest duration tested, implying that current investments in prevention can yield an economic return even far in the future. The Tarrant County pulmonary studies occurred only with patients who had received at least 20 weeks of treatment, so it is unknown how many members of the cohort died before that point. To estimate this number, we used a hypothetical death toll based on the known national death rate. This may not reflect the actual number of deaths in Tarrant County during this time.

Excessive deaths soon after treatments have been reported before now, but estimating such numbers is difficult, so we did not include such estimates in these analyses [34]–[36]. There have been no long-term studies of mortality among PTB patients. However, lung injury owing to other illnesses is associated with higher mortality rates [37]–[39]. Studies of the effect of pulmonary impairment on mortality have found significantly increased risk of death for those with moderate or severe impairment (1.6 and 2.7 hazard ratios, respectively). It is plausible that the cohort in this study would suffer similar mortality effects from PIAT [40]. Omitting the possibility of an increase of mortality in patients cured of TB might lead to an underestimate of QALYs lost to PTB. This, however, would result only in a change of the magnitude, not the direction, of our findings.

These measurements may not capture all health quality loss, as survivors of acute respiratory illness have been found to suffer impairment in general mood, cognition, and social functioning, as well as respiratory symptoms. Extra-pulmonary TB may also cause impairment through its effects on the brain and other organs [37]–[40]. Basing our estimates on the expected natural life of a normal cohort and not considering unknown factors such as possibly excessive risk of death, might underestimate the differences in QALYs lost. This makes our results a conservative estimate.

These findings give insight into the relative efficiencies of emerging technologies related to TB. Currently, several interventions are close to implementation. These technologies include those that shorten diagnosis and treatment of LTBI such as gamma interferon release assays for diagnosis, and isoniazid and rifapentine for treatment. Similarly moxifloxacin containing regimes shorten treatment of active TB. Our data show that any interventions resulting in more persons completing LTBI therapy will save 1.39 QALYs per case of TB averted; a strong argument for expansion of groups and age ranges considered appropriate for prophylactic treatment of LTBI. In contrast, interventions for shortening treatment of TB would result in little QALY savings. For example, reducing the duration of treatment for cure by 50% saves only 0.023 QALYs per illness. In addition, shorter treatment will not reduce the chronic pulmonary impairment associated with TB [10], [34]. These data illustrate that the greatest health savings will be achieved through strategies to prevent TB from developing, rather than strategies to shorten treatment once it has developed.

TB results in QALY loss because of illness, chronic impairment, and death—yet TB is a preventable disease. The current TB elimination strategy in low-incidence areas emphasizes case prevention through treatment of LTBI, but does not consider the value of potential savings from preventing chronic impairment in treatment recommendations [41]–[43]. Recognition of these benefits of treatment of LTBI may result in a number of changes. These changes include improved patient and clinician acceptance of LTBI therapy and expanding the population for whom LTBI treatment is recommended. In addition, these data provide additional rationale for support of TB control measures.

## Supporting Information

Abstracto S1 (0.03 MB DOC)
