# Supplementary material for: Personal and Societal Health Quality Lost to Tuberculosis
Source: PLoS One. 2009 Apr 8;4(4):e5080. doi: 10.1371/journal.pone.0005080 (PMC2660416; doi:10.1371/journal.pone.0005080)
Supplement: Abstracto S1 — (0.03 MB DOC) [file pone.0005080.s001.doc]

**Antecedentes**

En países desarrollados la tuberculosis es considerada una enfermedad con poca pérdida de Calidad de Vida Ajustada en Años (QALYs)*. El tratamiento de la tuberculosis es predominantemente ambulatorio y la muerte por esta causa es rara. Estudios de investigación han demostrado que hay secuelas pulmonares crónicas en la mayoría de los pacientes que han completado el tratamiento para la tuberculosis pulmonar (TBP). Estas secuelas y otros efectos de la tuberculosis en la calidad de salud no han sido considerados para la Calidad de Vida Ajustada en Años (QALYs)*. Por consiguiente tanto el daño causado por la tuberculosis en el individuo y el valor de la prevención en tuberculosis en la sociedad han sido subestimados. En el presente estudio estimamos el valor (QALYs)* en los pacientes con tuberculosis pulmonar (TBP) y a la vez describimos la pérdida de salud que prevalece en la Tuberculosis.

**Metodología/ Principales Resultados**

Nosotros calculamos los valores en salud durante la enfermedad y tratamiento, el daño pulmonar posterior a la tuberculosis (PIAT)**, las tazas de mortalidad, los años de pérdida de vida por muerte y la salud de la población normal. Posteriormente nosotros comparamos el tiempo de vida esperado en (QALYs)* en pacientes con Tuberculosis Pulmonar con un grupo de pacientes con la enfermedad latente y con un grupo de pacientes sin la infección. Pacientes con tuberculosis confirmadas por cultivo, demostraron menor tiempo de vida (QALYs)* comparados con aquellas sin tuberculosis. Los costos de la morbilidad en la tuberculosis aguda son 0.046 (QALYs)* ( 4% del total) por cada individuo. Para la morbilidad crónica los promedios calculados fueron de 0.96 (QALYs)* (78% del total). Los cálculos para la mortalidad fueron del 18% de la pérdida de Calidad de Vida Ajustada en Años (QALYs)*. El beneficio total para la sociedad por evitar un caso de PTB fue alrededor de 1.4 (QALYs)*.

**Conclusiones /Importancia**

La tuberculosis siendo una enfermedad prevenible, produce una perdida de la Calidad de Vida Ajustada en Años (QALYs)*. Esta perdida es debida en su mayoría al daño producido posterior a la cura microbiológica. Esfuerzos en prevenir la transmisión de la TB son traducidos en mejor calidad en salud. Estos esfuerzos en prevención deben ser prioridad por parte del sistema de salud.

*QALY:QualityAdjusted Life Years (siglas en Ingles)

** PIAT : Pulmonary impairment after tuberculosis
